# In Vivo Vasospasm Induction by Ultrasound Application in the Chicken Chorioallantoic Membrane Model

**DOI:** 10.1007/s12975-021-00960-y

**Published:** 2022-01-21

**Authors:** Katja Döring, Henning Schroeder, André Fischer, Swetlana Sperling, Milena Ninkovic, Christine Stadelmann, Dorothee Mielke, Veit Rohde, Vesna Malinova

**Affiliations:** 1 Department of Neurosurgery, University Medical Center Göttingen, Robert-Koch-Straße 40, 37075 Göttingen, Germany; 2 Department for Epigenetics and Systems Medicine in Neurodegenerative Diseases, German Center for Neurodegenerative Diseases, Göttingen, Germany; 3 Department of Neuropathology, University Medical Center Göttingen, Göttingen, Germany

**Keywords:** Chorioallantoic membrane model, Sonication, Vasospasm, In vivo assessment

## Abstract

Cerebral vasospasm is a highly investigated phenomenon in neurovascular research. Experimental vasospasm models are irreplaceable for the evaluation of new antivasospastic drugs. In this study, we assessed the reliability of in vivo vasospasm induction by ultrasound application in the chicken chorioallantoic membrane (CAM) model. After incubation of fertilized chicken eggs for four days, a fenestration was performed to enable examination of the CAM vessels. On the thirteenth day, continuous-wave ultrasound (3 MHz, 1 W/cm^2^) was applied on the CAM vessels for 60 s. The ultrasound effect on the vessels was recorded by life imaging (5-MP HD-microscope camera, Leica®). The induced vessel diameter changes were evaluated in a defined time interval of 20 min using a Fiji macro. The vessel diameter before and after sonication was measured and the relative diameter reduction was determined. A first reduction of vessel diameter was observed after three minutes with an average vessel-diameter decrease to 77%. The maximum reduction in vessel diameter was reached eight minutes after sonication with an average vessel diameter decrease to 57% (mean relative diameter reduction of 43%, range 44–61%), ANOVA, *p* = 0.0002. The vasospasm persisted for all 20 recorded minutes post induction. Vasospasm can be reliably induced by short application of 3 MHz-ultrasound to the CAM vessels. This might be a suitable in vivo model for the evaluation of drug effects on vasospasm in an experimental setting as intermediary in the transition process from in vitro to in vivo assessment using animal models.

## Introduction

Cerebral vasospasm is a frequent radiologic finding in the context of aneurysmal subarachnoid hemorrhage (aSAH), characterized by a delayed prolonged vasoconstriction, which is strongly associated with the occurrence of delayed infarction after aSAH. This phenomenon has been shown to be mediated by, e.g., endothelin-1 overproduction, nitric oxide underproduction, inflammation, and/or cortical spreading depression among other factors [1, 2]. Although the most recent research has revealed multiple factors potentially leading to ischemic complications following aSAH, severe vasospasm remains a major contributor to delayed cerebral ischemia that has to be considered during the management of patients with aSAH [2–4]. Unfortunately, several randomized controlled trials evaluating potent vasodilators could not demonstrate an improvement of patients’ outcome despite of showing a reliable resolution of moderate to severe angiographic vasospasm in aSAH patients [5–8]. Systemic side effects like pulmonary edema and hypotension might have at least partly contributed to these negative results, directing the scientific efforts to the development and evaluation of alternative drug delivery pathways such as intrathecal nanodrugs and to investigation of new anti-vasospastic drugs with a better risk profile [9]. Different SAH animal models have been already used for the assessment of vasospasm and for the evaluation of antivasospastic drugs [10–13]. However, the currently available SAH-models are exhibiting a varying vasospasm incidence and vasospasm severity. Furthermore, animal SAH models with high vasospasm incidence are also associated with high mortality rate [10]. Especially during the early developmental process of antivasospastic drugs, an alternative in vivo model to study the changes of vessel diameter would be of great relevance in order to reduce the use of animals for these purposes. The chicken chorioallantoic membrane (CAM) model exhibits high vascularity, and hence, has been proposed as an in vivo alternative to animal models for the assessment of vasoreactivity [14]. Furthermore, the CAM vessels, which are surrounded by a fluid environment in the allantoic cavity, seem to share common features with cerebral vessels within the subarachnoid space [15]. A CAM SAH-model has been developed through a CAM vein puncture, which led to histologically verified vasospasm of the umbilical artery occurring on day 3 and reaching its peak on day 5–7 after hemorrhage [15]. However, in this previously published study, vasospasm was histologically evaluated after sacrificing the eggs and an in vivo assessment of the vessel diameter changes was not reported. Ultrasound is widely used in medicine for diagnostic and therapeutic reasons. Several tissue effects of ultrasound including vessel contraction have been described in previous experimental studies [16, 17]. A contraction of the carotid artery could be demonstrated after continuous wave sonication for four minutes using a 3-MHz ultrasound probe [16]. The aim of this study was to combine ultrasound with the CAM model and to evaluate the possibility of vasospasm induction by ultrasound application in the CAM vessel model, which might serve as a reliable and easy to perform alternative to SAH animal models for in vivo vasospasm assessment.

## Materials and Methods

### Preparation of the CAM Model

**Fig. 1:**
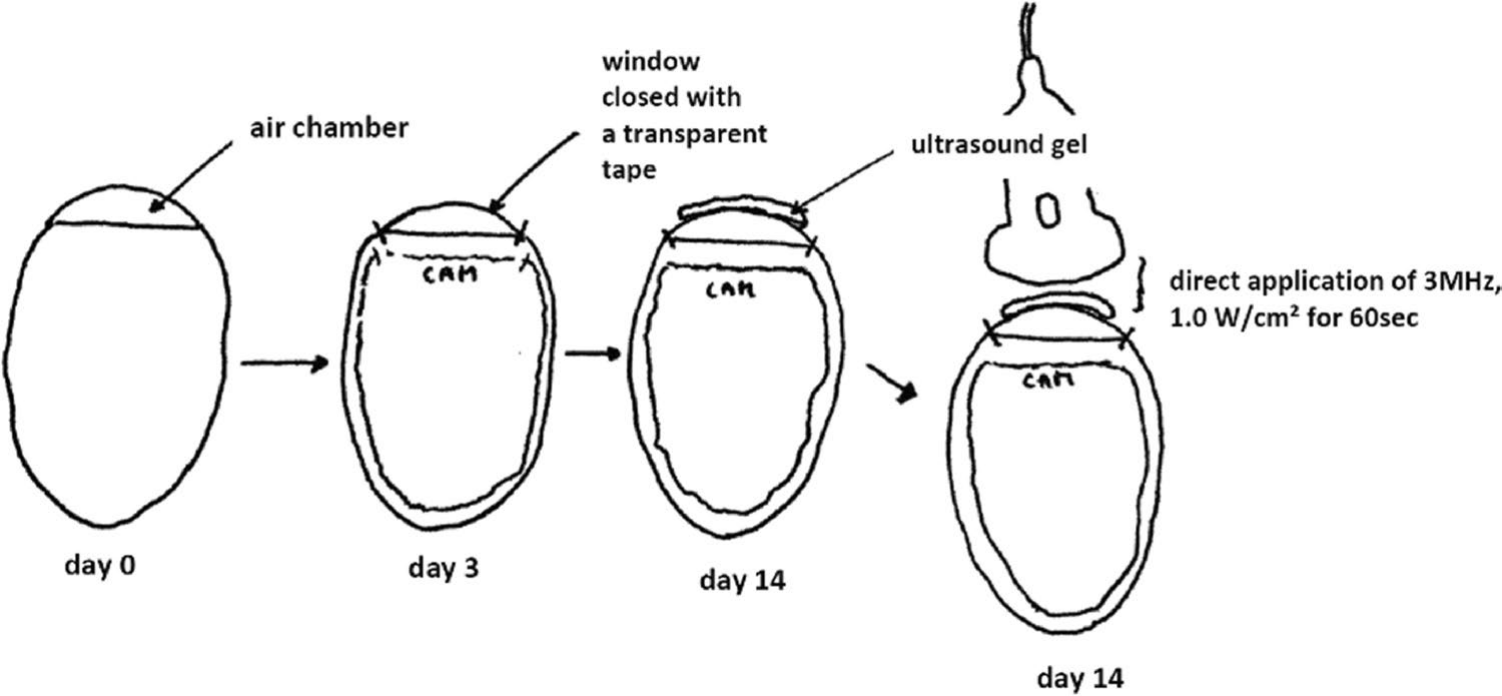
Chronological sequence of preparation of the fertilized eggs

The experiments were performed on 10 thirteen-day-old chicken embryo eggs. White fertilized eggs of the Bresse Gauloise chicken breed (Poulets-de-France, Hildesheim, Germany) were used, whose vascular CAM has been shown to be best seen under light [18]. A fortnightly incubation was ensured by the incubator BSS 420, species number 8301/01 (Grumbach® Automatic System GmbH & Co.KG, Nürnberg, Germany). The eggs were incubated at 37.7 °C with a constant humidity of 65%, then opened and windowed on the fourth incubation day by using a previously described technique [18]. For this purpose, the shell of the egg was stabilized at the lower pole, in the area of the air chamber, using Leukotape® (BSN medical, Hamburg, Germany), and then first penetrated with a small needle and then, starting from there, a round window was opened using scissors. The egg was securely sealed with transparent tape (Parafilm® Pechiney Plastic Packaging, OH, USA) until day 14 when the experiments were carried out. Before the ultrasound application to the CAM was started, the eggs were stabilized in a cup and, the CAM was protected by covering it with a parafilm (Parafilm® Pechiney Plastic Packaging, OH, USA). The sonication was performed by applying thermostable ultrasound gel (Sonosid® Asid Bonz GmbH, Herrenberg, Germany) on top of the parafilm and attaching the ultrasound applicator to it for a defined period of time (illustrated in Fig. 1**)**.

### Vasospasm Induction by Ultrasound Application

The in vivo vasospasm induction in the CAM model was based on the application of ultrasound to the CAM vessels. The specifications of the used ultrasound (Physiomed® Elektromedizin AG, Paderborn, Germany) were as follows: maximum effective area of the ultrasound probe was 5 cm^2^; the frequency was 3 MHz with continuous wave mode and a power density of 1 W/cm^2^. On day 14 after the incubation, ultrasound was applied to the CAM vessels for 60 s.

### Qualitative and Quantitative In Vivo Assessment of Vasospasm

**Fig. 2:**
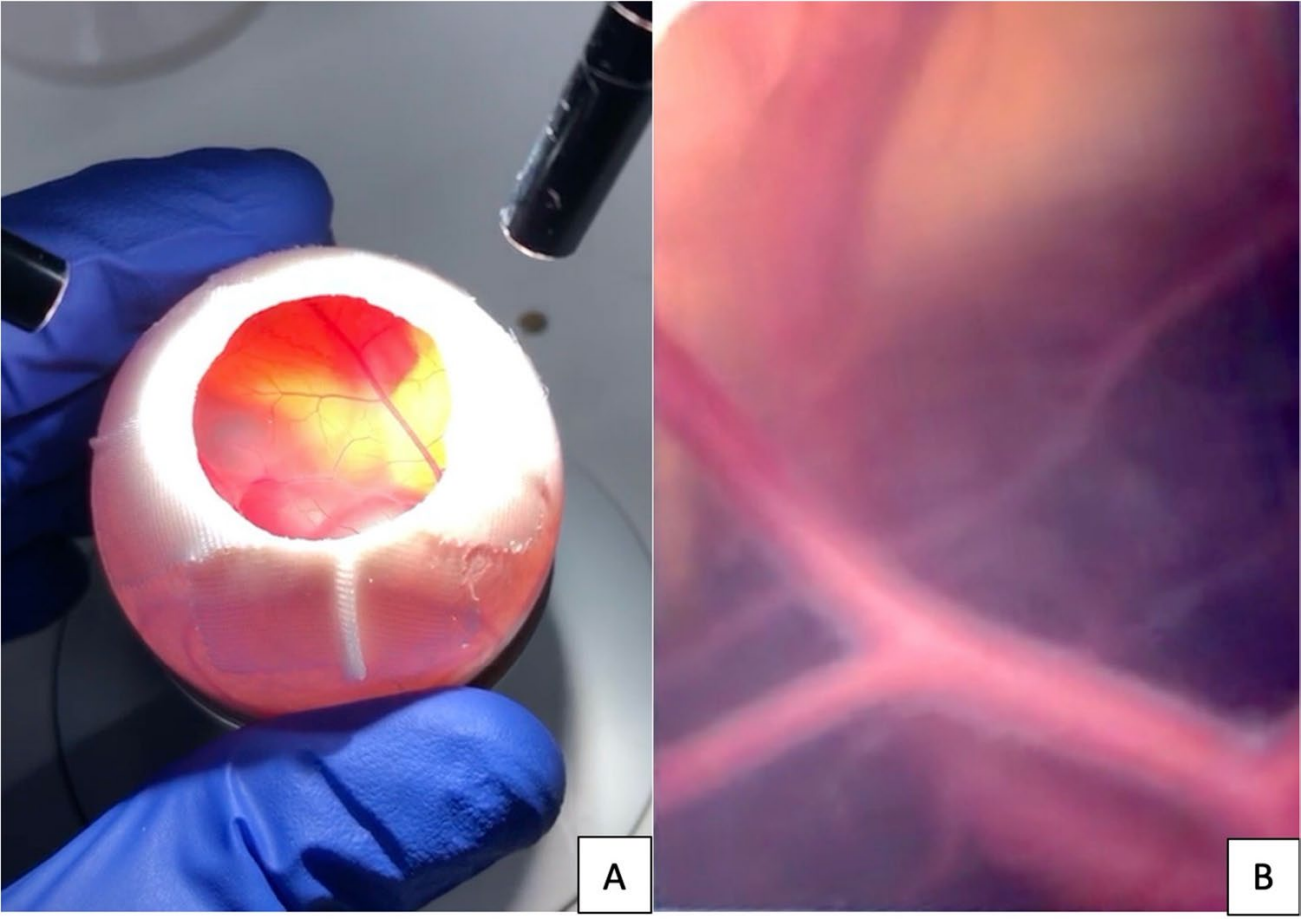
Prepared egg with exposed CAM vessels before ultrasound treatment (**A**), and with larger magnification under the microscope (**B**)

The visualization of the vessel diameter changes over time at the CAM was performed using a 5-megapixel HD-microscope camera (Leica® MC170 HD). The vessel diameter was determined as a quantitative measure at predefined time points using Fiji Image J [19], and expressed as arbitrary units, according to previously defined standards [19]. The initially measured vessel diameter before sonication was set to 100% and the relative reduction of the vessel diameter over time was determined. Images were taken at one-minute intervals over a period of 20 min and the macroscopic changes were recorded. The measurements were taken on only one vessel per CAM, reproducibly at the same location each time. The vessel measured over time was selected based on the macroscopic impression of the most pronounced vasospasm. Furthermore, we evaluated if other macroscopically visible responses to the ultrasound occurred such as bursting of vessels, changes within the capillary network, or changes in embryonic motility. Figure 2 shows the prepared egg with exposed CAM vessels before ultrasound treatment (A), and with larger magnification under the microscope (B). Additionally, vasospasm was induced in 6 eggs followed by an observational period of 24 h with vasospasm assessment after 30 min, 60 min, 2 h, and 24 h with the aim to the spontaneous vasospasm course after a longer time-period.

### Histological Assessment

In order to evaluate a possible permanent damage of the vessel wall through the sonication, histological analysis was performed 1 h and 24 h after ultrasonic vasospasm induction, respectively. For these reasons, hematoxylin eosin (HE) staining and terminal deoxynucleotidyl transferase dUTP nick end labeling (TUNEL) staining of the CAM were conducted using 6 eggs per group. The eggs were sacrificed 1 h and 24 h after vasospasm-induction by injecting 4% formaldehyde (MERCK, Darmstadt, Germany) directly under the allantoic cavity. Following the CAM dissection, the samples were fixated in 4% formaldehyde and stored at 4 °C. After dehydration and clarification, the CAM samples were than embedded in paraffin blocks and cut into 3 µm thick slices using a Leica RM2135 rotary microtome (Leica, Wetzlar, Germany). A TUNEL-staining was performed using DeadEnd™ Fluorometric TUNEL kit (Promega, Walldorf, Germany) to assess whether apoptosis within the vessel wall happened. For this purpose, paraffin-embedded blocks were dewaxed and rehydrated in decreasing concentrations of ethanol (100%, 95%, 85%, 70%, 50%). Further wash cycles included immersion in 0.85% sodium chloride and phosphate-buffered saline solution for 5 min each. For antigen retrieval, the sections have been incubated in 100 µl of a 20 µg/ml proteinase K for 8 min at room temperature. The next steps included washing and fixing again according to the manufacturer’s instructions. After equilibration, 50 µl of the reaction mixture was now applied to the tissue. The slides were then stored for 60 min at 37 °C in a humidified chamber. To stop the reaction, the slides are immersed in 2 × saline sodium citrate for 15 min. The green fluorescence of apoptotic cells (fluorescein-12-dUTP) was detected in a red background of propidium iodide (MERCK, Taufkirchen. Germany) used as a counterstain. Microscopic analysis was performed by using the Leica DMi8 microscope (Leica, Wetzlar, Germany).

### Statistical Analysis

Statistical analysis was performed using the statistical program GraphPad Prism (Version 8, GraphPad Software, San Diego, CA, USA). Descriptive statistics were applied to determine the vessel diameter changes. One-way ANOVA analysis was used to evaluate differences in vessel diameter at predefined time points.

## Results

All incubated eggs have developed in time and could be included in the study. The preparation of the model has proved to be safe and reproducible in all 10 eggs without complications.

### Vasospasm Induction

**Fig. 3:**
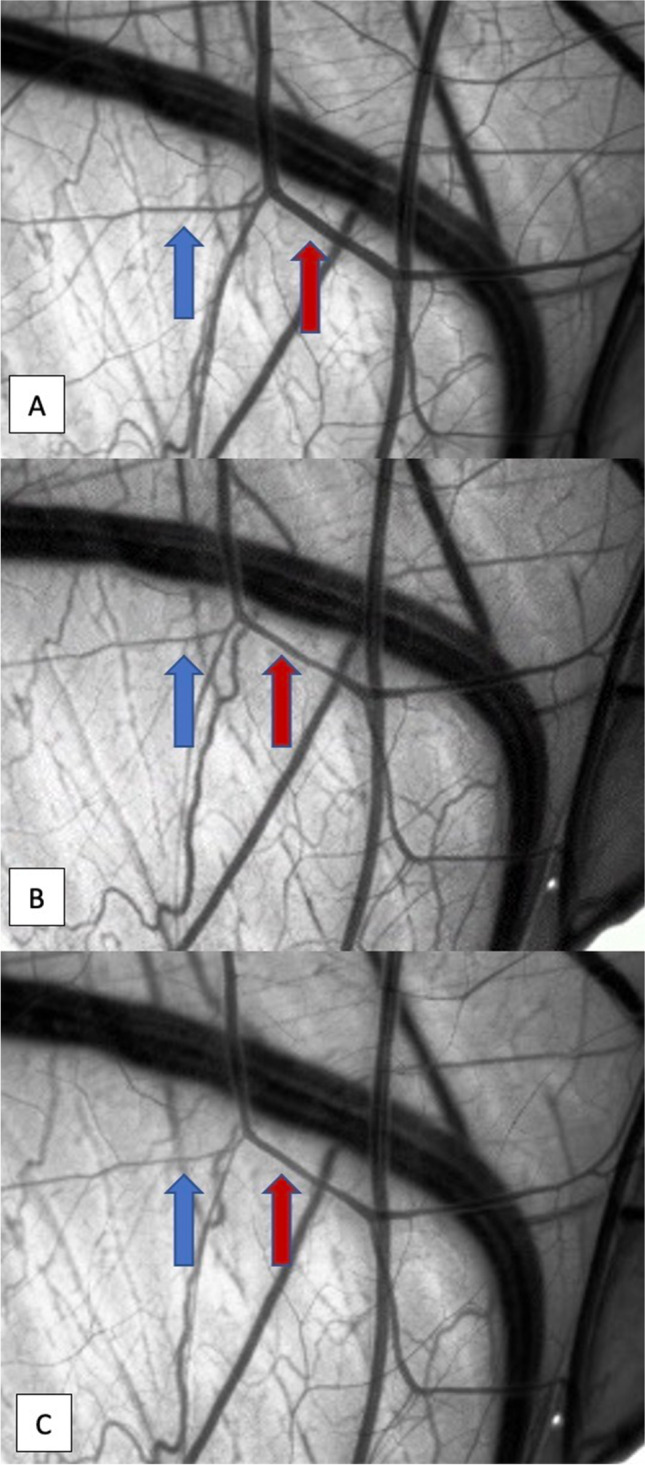
Visualization of the vasospasm development after ultrasound application for 60 s. (**A**) Vessel diameter before the ultrasound application; (**B**) first vessel diameter reduction 3 min after ultrasound application; (**C**) maximal vessel diameter reduction 8 min after ultrasound application

**Fig. 4:**
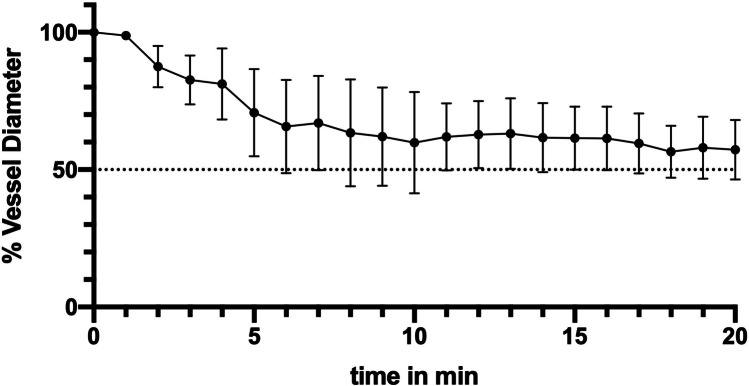
Time course of vessel diameter changes after vasospasm induction (mean ± SD) over the observation time of 20 min

The mean initial vessel diameter was 0.21 arbitrary unit ranging from 0.08 to 0.36, which was considered 100%. A first significant reduction of vessel diameter was observed after three minutes with an average vessel diameter decrease to 77% (mean relative diameter reduction of 23%, range 58–88%). The vasospasm development over time is demonstrated in Fig. 3. The maximal reduction in vessel diameter was reached eight minutes after ultrasound application with an average vessel diameter decrease to 57% (mean relative diameter reduction of 43%, range 44–61%), ANOVA, *p* = 0.0002 (Fig. 4). The vasospasm severity significantly differed between the CAMs ranging from 19 to 83% relative vessel diameter reduction 8 min after sonication (ANOVA, *p* < 0.0001). A spontaneous vasospasm resolution starting 1 h after vasospasm induction was observed reaching 82% of the former vessel diameter by 2 h, and 93% of the initial vessel diameter 24 h after the ultrasonic vasospasm induction.

### Macroscopic Changes

An increase in embryonal motility was observed in all eggs after ultrasound treatment. Furthermore, a reduction of the capillary network was observed. An injury of the vessels and the capillary network in the sense of blood leakage could not be seen.

### Histological Analysis

**Fig. 5:**
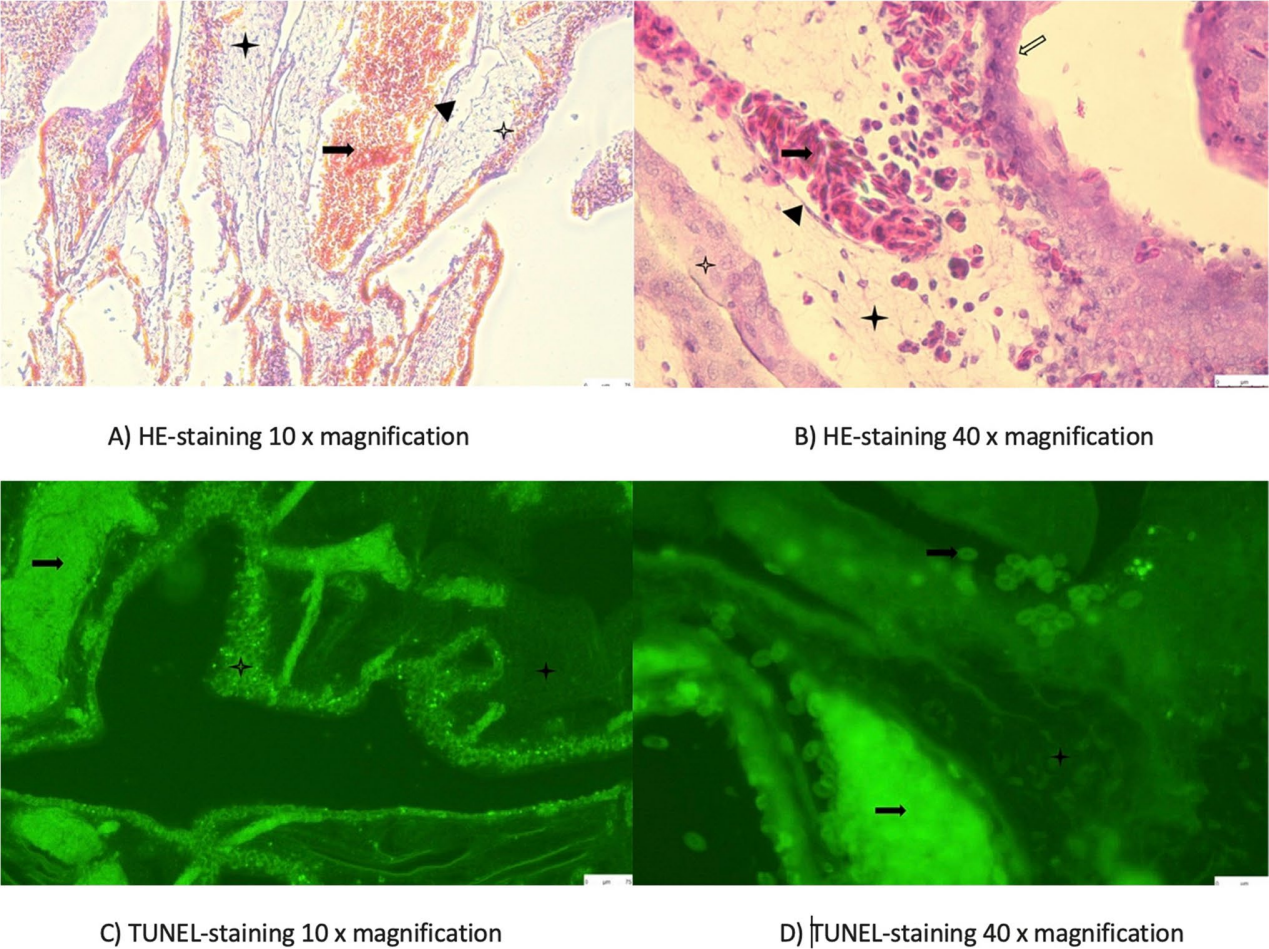
HE-staining (**A** and **B**), and TUNEL-staining (**C** and **D**) of the CAM 1 h after ultrasonic vasospasm induction showing no apoptosis within the vessel wall.  Endothelium,  Erythrocytes,  Mesenchymal layers,  Chorion,  Allantois

**Fig. 6:**
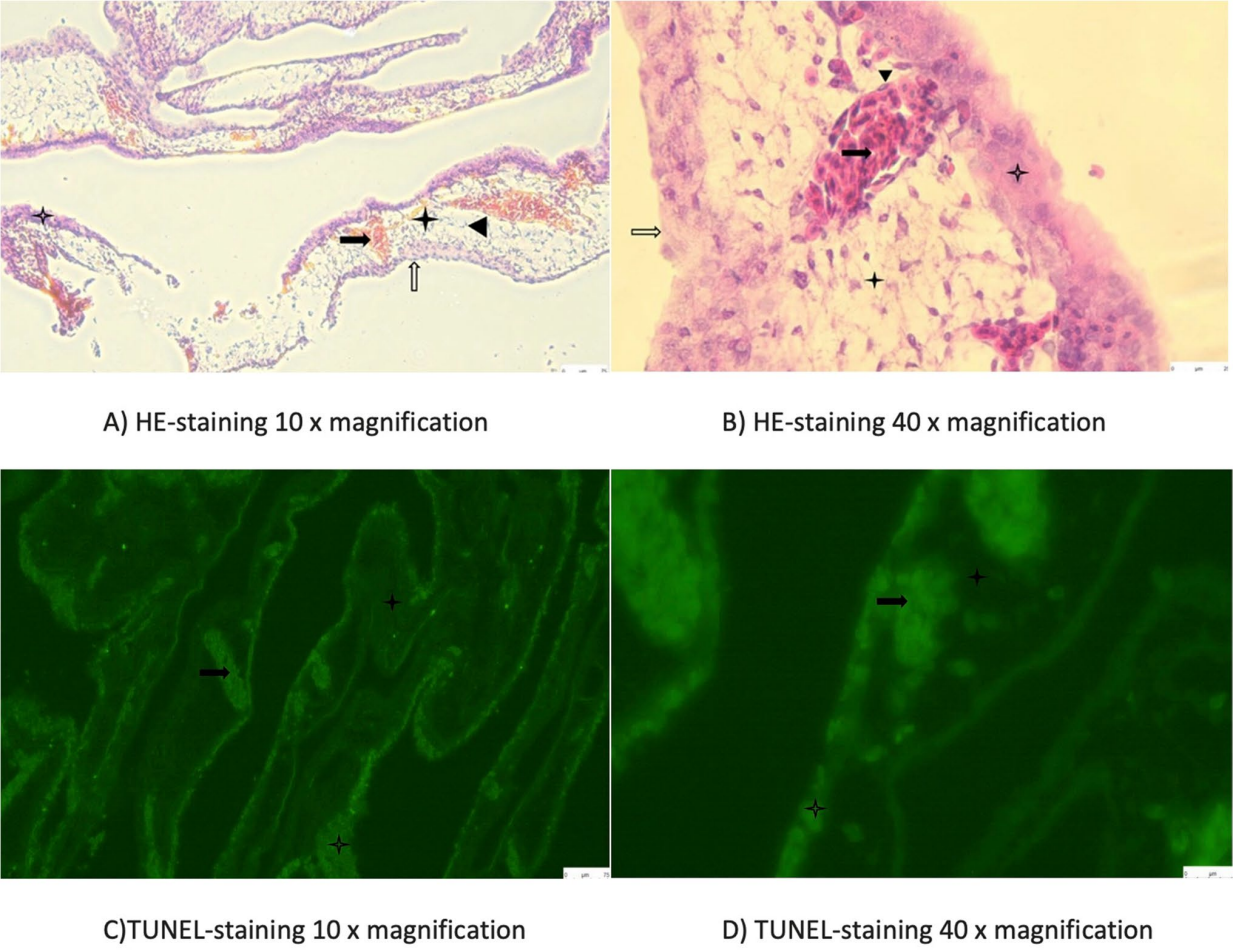
HE-staining (**A** and **B**), and TUNEL-staining (**C** and **D**) of the CAM 24 h after ultrasonic vasospasm induction showing no apoptosis within the vessel wall.  Endothelium,  Erythrocytes,  Mesenchymal layers,  Chorion,  Allantois

The histological analysis did not show damage or apoptosis within the vessel wall neither 1 h nor 24 h after the ultrasonic vasospasm induction. An example of the HE- and TUNEL-staining after 1 h and 24 h is demonstrated in Fig. 5 and Fig. 6, respectively.

## Discussion

In this experimental study, we described a technique for vasospasm induction by applying continuous wave ultrasound in the chicken CAM model for in vivo vasospasm assessment. A reliable vasospasm induction could be observed in all embryo eggs with an average vessel diameter reduction of 40–60% eight minutes after applying ultrasound for only 60 s. The vasospasm development could be directly recorded positioning a microscope camera over the exposed CAM. The severity of vasospasm varied between the CAMs enabling an evaluation of different vasospasm severity grades. The in vivo ultrasonic induced vasospasm model in the CAM vessels is characterized as a reversible significant vasoconstriction of the sonicated CAM vessels, which could be reliably induced after ultra-short sonication of the vessels, and reliably persisted for at least 1 h and resolved spontaneously starting 1 h after sonication and reached the former vessel diameter after 24 h again. This model does not resemble the plethora of pathophysiological processes leading to vasospasm after SAH, but the final product of significant vasoconstriction providing an in vivo possibility to study anti-vasospastic drug effects for treatment of vasospasm as an intermediary in the transition process from in vitro evaluation to in vivo assessment using animal models. During the early stages of antivasospastic drug development in particular, this model could be of great advantage and might reduce the number of animals needed to be operated on for an in vivo vasospasm induction.

### Ultrasound-Mediated Effects

The exact mechanisms behind the induction of vasoconstriction by ultrasound application have not been determined yet. A complex interplay between the endothelial cells lining the vessel wall and the smooth muscle cells of the underlying tissue determines the vascular tone. This cellular interaction depends on a wide range of signals, including free radicals such as nitric oxide (NO) that are exchanged between the two cell populations and on mechanical forces that are transduced mainly by the endothelial cells [17]. Frequency-dependent, ultrasound-induced changes in vascular tone have been described in the literature. While low-frequency ultrasound (20 kHz) has been shown to cause dilatation of blood vessels in vitro [16], in vitro application of ultrasound in the MHz frequency range on the carotid artery resulted in vasoconstriction [16]. The effect was reversible and lasted for several hours leading to the assumption that ultrasound might not cause long-lasting damage on the vessel wall. According to existing literature, the ultrasound-induced effects on tissue can be generally divided into thermal and non-thermal mechanisms [20, 21]. Three factors have been reported to possibly play a role in the processes of vascular tone changes induced by ultrasonic wave: fluid mechanical shear stress caused by fluid streaming, radiation forces, and local heating [16, 22]. It is assumed that small vessels such as arterioles with a higher proportion of smooth muscle in their walls might show a stronger reaction to ultrasound [16]. When an ultrasound wave propagates in tissue, a mechanical strain is induced resulting in relative change in dimensions or shape of the body that is subjected to stress [21]. A rarefaction of the capillary network as well as changes in vessel shape were observed microscopically in our study, especially in small vessels, which might represent an indirect sign for capillary constriction of small vessels induced by ultrasound, making these vessels macroscopically invisible. The observations in our study also support the assumption that the intensity of the ultrasound-induced effects is dependent on the vessel size in an inversely proportional manner. Considering this, the CAM model seems to be mainly suitable for assessment of vasospasm within the microcirculation. Both thermal and non-thermal effects might have contributed to the observed effects in our study. Since the focus of this study was put on the feasibility of the technique rather than on the molecular mechanism behind it, we cannot provide further information to clarify this question. Future targeted measurements of thermal and non-thermal ultrasound effects are required to shed light on this subject. In this experimental setting, we were not able to perform quantitative measurements of thermal or mechanical effects, due to methodological limitations. Ultrasonic dosimetry would be necessary to determine the ultrasonic energy and to evaluate its interaction with biological materials. The most widely used quantity in ultrasonic bioeffect and biophysical studies is ultrasound intensity. In our study, we applied ultrasound with the intensity of 1 W/cm^2^ and did not observe any side effects. The prolonged observation of the eggs following the ultrasonic vasospasm induction for a time-period of 24 h showed a spontaneous vasospasm resolution starting after 1 h and reaching 93% of the initial vessel diameter again after 24 h, confirming a reversible vasospasm induction. The histological analysis of the CAM 1 h and 24 h after ultrasonic vasospasm induction showed no apoptosis within the vessel wall, which is also indication that no permanent damage of the vessel occurs during the ultrasonic vasospasm induction. Further experiments and histological analyses are necessary to elucidate the mechanisms underlying ultrasound-induced vasospasm in the CAM model.

### Advantages and Shortcomings of the Ultrasound-Induced Vasospasm CAM-Model

Since the ultrasound-induced vasospasm CAM model is an easy to perform technique with the possibility of reliable in vivo vasospasm induction, this model allows an instant assessment of drug effects on vasospasm, which represents its main advantage. The findings in the CAM model would allow a better planning of further animal experiments and, therefore, allow a reduction of animals needed to be included into the experiments according to the 3R-principle (reduce, refine, replace). A further advantage is the fact that vasospasm could be induced within a short period of time. Compared to animal experimental models, the CAM model is also more cost-effective. On the other side, this model has clear limitations for the experimental assessment of early and delayed brain injury, wherefore established animal SAH-models remain irreplaceable [10]. Animal SAH-models are providing a more realistic representation of the processes happening after SAH allowing an assessment of the pathophysiology behind these processes. The ultrasonic vasospasm induction in the CAM model does not represent the pathophysiologic processes behind aSAH or other clinical conditions associated with vasoconstriction of cerebral vessels, hence, does not allow assessment of underlying pathomechanisms, which is a limitation of the model. The CAM model should be regarded as reliable, fast, and rather simple model of in vivo vasospasm induction allowing a pre-evaluation of antivasospastic drugs in an experimental in vivo setting before the initiation of further assessment in animal models, which might be of advantage during the translational process from in vitro to in vivo developmental steps of new antivasospastic drugs.

## Conclusion

Vasospasm can be reliably induced by direct short application of 3 MHz-ultrasound to the CAM vessels. This might be a suitable in vivo model for the evaluation of drug effects on vasospasm in an experimental setting and could serve as valid intermediary during the transition process from in vitro evaluation to in vivo assessment using animal models.

## Data Availability

All relevant data and materials are presented in the manuscript.
